# Varietal Susceptibility of Olive to *Pseudomonas savastanoi* pv. *savastanoi* and the Antibacterial Potential of Plant-Based Agents

**DOI:** 10.3390/microorganisms12071301

**Published:** 2024-06-26

**Authors:** Laura Košćak, Janja Lamovšek, Marina Lukić, Tvrtko Karlo Kovačević, Edyta Đermić, Smiljana Goreta Ban, Nikola Major, Sara Godena

**Affiliations:** 1 Department of Agriculture and Nutrition, Institute of Agriculture and Tourism, Carlo Hugues 8, 52440 Poreč, Croatia; laura@iptpo.hr (L.K.); marina@iptpo.hr (M.L.); tvrtko@iptpo.hr (T.K.K.); smilja@iptpo.hr (S.G.B.); nikola@iptpo.hr (N.M.); 2 Agricultural Institute of Slovenia, Hacquetova ulica 17, 1000 Ljubljana, Slovenia; janja.lamovsek@kis.si; 3 Department of Plant Pathology, Division of Phytomedicine, University of Zagreb Faculty of Agriculture, Svetošimunska Cesta 25, 10000 Zagreb, Croatia; edermic@agr.hr

**Keywords:** olive knot disease, olive genotype susceptibility, phenols, plant-based antibacterial agents, strain virulence

## Abstract

Olive knot disease, caused by the bacterium *Pseudomonas savastanoi* pv. *savastanoi*, causes great damage in olive orchards. While control measures of *P. savastanoi* pv. *savastanoi* in olive orchards primarily rely on pruning and copper-based treatments, the use of antibiotics as bactericidal preparations in agriculture is limited and highly restricted. However, plants are naturally endowed with protective molecules, such as phenolic compounds, which defend them against herbivores, insects, and microorganisms. This research aimed to test the virulence of five strains of *P. savastanoi* pv. *savastanoi* isolated from different growing regions and olive varieties, and to examine whether there is a difference in plant susceptibility based on the variety. An additional goal was to test the antimicrobial activity of olive mill wastewater, known for its high content of phenolic compounds, and aqueous garlic hydrolysate, as well as to compare them with a commercial copper-based product, pure hydroxytyrosol, and a standard antibiotic as references. Analysis of knot characteristics showed variations in the virulence of the *P. savastanoi* pv. *savastanoi* strains, with the highest virulence being observed for the strain I7L and the lowest virulence for the strain B45C-PR. The olive cultivar Rosinjola displayed higher susceptibility compared to Frantoio, Buža, and Leccino, while cv. Istarska bjelica exhibited the least susceptibility compared to the other investigated olive cultivars. In an attempt to explore alternative solutions for disease control, in vitro tests revealed that the phenol HTyr, GE, and the wastewater with the highest total phenolic content (cv. Istarska bjelica) possess the highest antibacterial activity. This supports the role of polyphenols in host defense, aligning with previous field observations of lower susceptibility of cv. Istarska bjelica to olive knot disease. These findings highlight the complex nature of olive knot interactions with bacterial strains and olive cultivars, simultaneously accentuating and underscoring the importance of considering the host’s defenses as well as bacterial virulence in disease management strategies.

## 1. Introduction

One of the most persistent bacterial diseases worldwide, particularly prevalent in the Mediterranean region, is olive knot disease affecting olive trees (*Olea europaea* L.) [1,2,3]. This disease is caused by infection with the Gram-negative bacterium *Pseudomonas savastanoi* pv. *savastanoi*, and it is recognized as a chronic disease of olive trees with significant economic consequences, as it inflicts both direct and indirect damage in olive production [4]. In agricultural areas, the management of phytopathogenic bacteria predominantly relies on copper-based preparations. However, the improper usage of copper can lead to phytotoxicity and environmental contamination. Moreover, there is growing concern over the development of resistance to copper-based treatments, highlighted by findings indicating resistance among populations of *P. syringae* pv. *actinidiae* in kiwi orchards in Italy, Japan, and Korea, for example [5,6]. 

Given the limited choices regarding current preparations for controlling bacterial plant diseases and the reliance on phytosanitary measures, there is a pressing need to explore and assess alternative potential tools for effective plant protection management against bacterial diseases. The antibacterial efficacy of plant-based agents is thought to vary depending on the bacterial species or pathovar. Some studies have highlighted notable differences in the virulence of bacterial pathogens [7,8,9]. For example, research by Moretti et al. [9] mentioned variations in virulence among *P. savastanoi* pv. *savastanoi* strains, with Italian strains exhibiting higher virulence compared to those isolated from olive trees in the eastern coastal region of the South Adriatic, comprising parts of south Croatia, Bosnia and Herzegovina, and Montenegro. Moreover, the severity of damage caused by olive knot disease varies significantly depending on the olive variety [10,11,12,13,14], often resulting in the categorization of varieties into distinct clusters ranging from low to highly susceptible cultivars. 

Given the absence of bactericidal preparations for effective *P. savastanoi* pv. *savastanoi* control, a growing interest remains in identifying olive cultivars that exhibit tolerance to this pathogen as a potential solution. Despite extensive research into the susceptibility of numerous cultivars to olive knot disease, and recent research on some Croatian genotypes [15], the susceptibility of numerous autochthonous Croatian olive varieties is yet to be determined. Another potential solution for *P. savastanoi* pv. *savastanoi* control is the use of plant-based agents with potential antibacterial properties. In recent years, plant-based products such as essential oils and plant extracts have gained significant attention in the scientific community due to their well-established antibacterial properties [16]. Among these, garlic (*Allium sativum* L.) extract has been particularly highlighted for its antibacterial effects [17,18,19]. However, most studies have focused on its efficacy against human pathogenic bacteria, demonstrating potential for treating antibiotic-resistant bacterial strains in vitro. 

The antimicrobial potential of wastes of agricultural production, such as olive mill wastewater (OMWW), has been considered for evaluation against plant pathogens. OMWW is the liquid fraction of the residues after olive processing into oil, which contains polysaccharides, water-soluble carbohydrates, proteins, and lipids which, in addition with macro- and microelements, stimulate microbial growth. OMWW is also known for its phytotoxicity and antimicrobial effect based on the high concentration of hydrophilic phenolic compounds [20]. The disposal of raw OMWW poses an environmental hazard, often resulting in soil and water contamination when discarded in ponds, thereby increasing costs for proper management for farmers [21,22]. To mitigate this severe pollution, some researchers have focused on the recovery of OMWW, exploring its potential for different purposes, including as antimicrobial agents [23,24]. Notably, in these studies, the OMWWs obtained from different olive cultivars show differences in their effect against phytopathogens. It is hypothesized that variations in the chemical composition and concentrations of phenolic compounds in OMWWs are cultivar-dependent, leading to differences in their antimicrobial effects [21,24,25]. 

As it is known, phenols play a significant role in numerous physiological processes within plants, serving as key components in the structure of plant cell walls. Thus, phenolic compounds contribute to the plant defense mechanisms against pathogens [26]. It is well established that phenolic compounds exhibit antimicrobial properties [21], with studies indicating that OMWWs and plant extracts such as garlic extract are particularly effective in this regard [17,21,24]. The antimicrobial effects of these agents have been demonstrated against numerous bacteria, including *Staphylococcus aureus*, *Escherichia coli*, *Enterococcus faecalis*, *P. aeruginosa*, *Xanthomonas campestris*, among others [17,21,24,25]. The phenolic compound hydroxytyrosol found in OMWWs is of significant interest, highlighted by its non-toxic properties. However, conflicting findings among researchers regarding its efficacy in antibacterial studies have resulted in controversy surrounding its potential against pathogenic bacteria [27]. Moreover, limited information exists regarding its efficacy against plant pathogenic bacteria, and thus more coherent studies are needed to clear the questions of its use in plant protection formulations. 

Strains of *P*. *savastanoi* pv. *savastanoi*, originating from Italy, Croatia, and Slovenia, were selected for the following objectives: (i) to determine potential differences in virulence through an in planta experiment, (ii) to assess the susceptibility of three Croatian autochthonous olive cultivars (cv. Buža, Rosinjola and Istarska bjelica) to olive knot disease, and (iii) to explore variations in the antibacterial effects of plant-based preparations in vitro. These agents included OMWWs obtained from the processing of olives from five different cultivars, the phenolic compound hydroxytyrosol, and a fresh garlic hydrolysate. 

## 2. Materials and Methods

### 2.1. Preparation of Bacterial Inoculum

In this study, a panel of *P. savastanoi* pv. *savastanoi* strains consisting of three isolates (CFBP5075, A1-1 and I7L) for laboratory antimicrobial experiments and five *P. savastanoi* pv. *savastanoi* strains (CFBP5075, A1-1, I7L, P15N and B45C-PR) for greenhouse screening of olive susceptibility to olive knot disease were tested. The strains I7L (45°22′34″ N; 13°60′25″ E), P15N (45°17′12″ N; 13°39′56″ E) and B45C-PR (45°24′42.50″ N; 13°30′10.22″ E) were isolated from olive trees exhibiting symptoms of infection, specifically from the cultivars Leccino, Nocciara, and Porečka rosulja, respectively, within the Croatian Istria region. The *P. savastanoi* pv. *savastanoi* strain A1-1 (45°53′31.55″ N; 13°61′75.14″ E) was isolated from the Arbequina cultivar growing in an olive orchard in the Slovenian Istria region. The reference strain CFBP5075 (originating from Italy) was purchased from the National Institute of Agricultural Research (INRA, Paris, France). These strains were previously characterized according to the LOPAT scheme and molecularly identified as strains of *P. savastanoi* pv. *savastanoi* by Košćak et al. [28]. Stock cultures were prepared through sub-culturing on agarized King’s B (KB) growth medium, followed by incubation at 26–27 °C for 24–48 h [3]. 

For the determination of antimicrobials, the broth microdilution assay was accessed according to the Clinical and Laboratory Standards Institute guidelines [29]. Pure bacterial colonies of cultured bacterial strains were selected from the plates and transferred to liquid KB growth medium. These bacterial suspensions were incubated overnight in a shaker-incubator at 27 °C and 80 rpm. The density of the bacterial suspensions was adjusted to approximately 10^6^ CFU/mL using spectrophotometric measurements; specifically, it showed an optical density (OD_600_) of 0.12–0.15. 

### 2.2. Evaluation of Olive Knot Disease Susceptibility in Greenhouse Experiment

**Figure 1:**
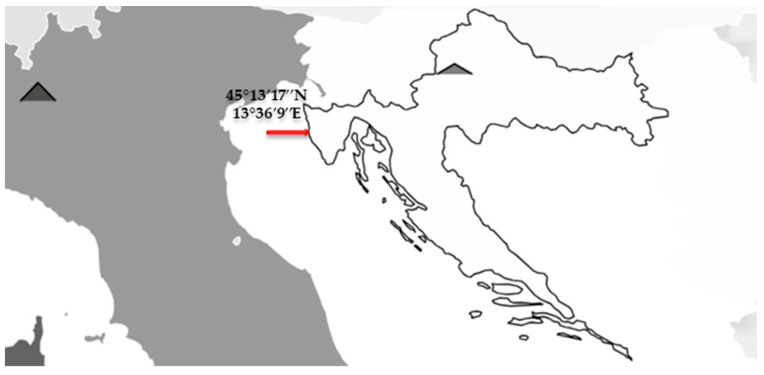
The location of conducted greenhouse experiment. Red arrow indicates location according to geographical coordinates.

The reference *P. savastanoi* pv. *savastanoi* strain CFBP5075 (Italy) and one Slovenian strain, A1-1, along with three Croatian strains labeled as I7L, P15N and B45C-PR, were subjected to assessment for their pathogenicity and the susceptibility of autochthonous Croatian olive varieties to them, namely Rosinjola, Buža and Istarska bjelica, as well as the Italian varieties Leccino and Frantoio (Figure 1). The greenhouse experiment was conducted at the Institute of Agriculture and Tourism, Poreč, Istria, Croatia (45°13′17″ N; 13°36′9″ E) (Figure 1), in March 2023, with irrigation included. Plants were irrigated using a drip irrigation system, ensuring that all plants received the same amount of water. The irrigation schedule was designed to maintain adequate soil moisture without causing waterlogging or drought stress. Plants were regularly monitored for signs of stress, and any issues observed were promptly addressed. 

**Figure 2:**
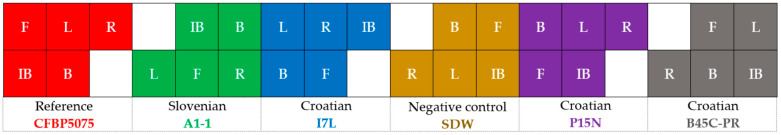
The experimental design for the olive knot disease susceptibility greenhouse experiment. Each *P. savastanoi* pv. *savastanoi* strain along with the negative control was inoculated with sterile distilled water (SDW) on 12 plants of each olive cultivar (IB—Istarska bjelica, L—Leccino, R—Rosinjola, B—Buža, F—Frantoio). Each colored square represents the combination olive cultivar × inoculation treatment.

The experiment in the greenhouse included a total of 360 olive plants, with 60 plants per cultivar. Each cultivar was subjected to 6 treatments, i.e., infection with five different *P. savastanoi* pv. *savastanoi* strains and one negative control (sterile distilled water) (Figure 2). Each combination olive cultivar × treatment was represented by 12 plants.

**Figure 3:**
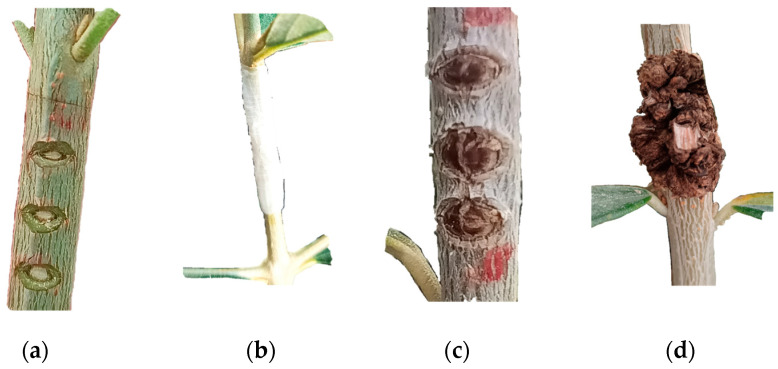
Steps of experimental inoculation of bacterium *P. savastanoi* pv. *savastanoi* for the determination of strain virulence and olive cultivar susceptibility to olive knot disease in the greenhouse experiment. (**a**) V-shaped fresh wounds on olive bark; (**b**) wounds covered with parafilm M after inoculation with bacterial suspension; (**c**) healed wound on olives 10–15 days after inoculation, and (**d**) developed symptoms of olive knot disease on the inoculation sites after six months of inoculation.

For this evaluation, bacterial suspensions containing approximately 10^8^ CFU/mL (McFarland 0.5 Standard) were utilized. Bacterial suspensions were inoculated into wounds in the bark of 1–2-year-old olive stems. Following the method described by Sisto et al. [11] (Figure 3), at five different places in the bark of each young plant (5 repetitions), three wounds were carved per plant (Figure 3a), resulting in separate knots (Figure 3c) or one clustered knot (Figure 3d) for each repetition. One cluster was measured as a single observation of knot development (the mean value if separated knots were present). Following wounding and inoculation with bacterial suspensions, the sites were covered with parafilm M (Figure 3b). The negative control comprised 12 plants of each variety, inoculated with SDW. Parafilm removal was performed after approximately 10–15 days (Figure 3c), following the observed formation of a callus of the wounds on the control (non-inoculated) plants. Initial symptoms appeared three months post-inoculation, with knot measurements conducted after six months in September 2023, coinciding with most of the plants developing knots at the inoculation sites (Figure 3d).

### 2.3. Preparation of Antibacterial Treatments

A total of 14 antimicrobial treatments were tested for their potential antibacterial effect against *P. savastanoi* pv. *savastanoi*: 10 samples of olive mill wastewater (OMWW), a water extract of garlic (GE), along with the solution of hidroxytyrosol in water (HTyr), the standard antibiotic tetracycline (AB), and a copper-based preparation (Cu) (Table 1). 

The OMWW samples utilized as antibacterial treatments were obtained from the olive oil mill situated in Istria after the two-phase olive processing carried out in the year 2021. Upon collection, the OMWWs originating from five distinct olive varieties were kept for nine days at a temperature of +4 °C [30], and then the OMWWs underwent filtration, involving centrifugation at 4000 rpm for 10 min at +4 °C, utilizing a Hettich 320 R centrifuge (Germany). Subsequently, the centrifuged treatments were divided into two fractions of a total volume of 4.5 L each. To protect the phenolic compounds present in the OMWWs against oxidative degradation, one fraction underwent treatment with an HCl solution, aiming to attain a final pH of 2.0 [24], while the second fraction remained as raw filtered OMWWs to test whether acidification as a pretreatment was necessary. The pH of OMWW samples was measured at room temperature with a pH meter (MP220 Basic pH/mV/°C Meter, Mettler-Toledo GmbH, Giessen, Germany) after calibration with pH buffers certified (Mettler-Toledo GmbH, Greifensee, Switzerland) as a reference material. Consequently, the OMWWs were categorized into two distinct treatment groups based on the presence or absence of HCl, as delineated in Table 1. Prior to analysis and application, the preparations were maintained at −20 °C until the antibacterial experiments. 

### 2.4. Determination of Total Phenolic Content

Total phenolic content (TPC) in OMWW samples was estimated based on the reaction of coloration of phenols with the Folin–Ciocalteu reagent (FCR) and sodium carbonate, following spectrophotometric reading of light absorbance at 725 nm (Carry UV/Vis 50, Varian Inc., Harbor City, CA, USA). The procedure was as follows: 1.0 mL of sample or diluted sample/standard solution/blank was diluted with 5 mL of water to which 0.5 mL of FCR was added and stirred. After exactly 3 min, 1 mL of sodium carbonate saturated solution (20 g/L) was added. The reaction mixture was stirred and left to stand for exactly 1 h until spectrophotometric reading. Gallic acid (GA) was used as a reference. The stock solution was prepared by weighing 0.1 g GA and diluted in 100 mL of water. A calibration curve was built with 10 standard solutions of GA prepared in 25 mL volumetric flasks by adding aliquots of GA stock solutions in the range from 0.125 to 2.000 mL in order to adjust concentrations to fit within the linear range of absorbance readings of the spectrophotometer (Abs = 0.4401 × Conc. + 0.1125; R^2^ = 0.9973). The results, corrected with the sample dilution inserted into the reaction, were expressed as GA equivalents (GAE) in one milliliter of OMWW (mg GAE/mL). Measurements were conducted in duplicate, and the results were reported as the average value of two measurements along with the standard deviation. Deionized water was obtained with the Elix 3 purification system (Millipore, Bedford, MA, USA). Ethanol, 96% p.a., was a product of Alkaloid Skopje (Republic of Macedonia). FCR and sodium carbonate were obtained from Kemika, Croatia. GA was a product of Sigma-Aldrich (Merck, Darmstadt, Germany). 

The garlic extract (GE) was prepared according to the methodology described by Bryan-Thomas et al. [31]. Briefly, 20 g of fresh garlic cloves were cut into small pieces (approximately 3 mm) and submerged in 100 mL of sterile distilled water in a glass covered with aluminum foil to protect it from potential light degradation. The top of the glass container was further sealed with parafilm M to prevent evaporation. The solution was allowed to stand for 24 h at room temperature, after which it was filtered twice through 0.22 μm sterile antibiotic filters using a syringe. The TPC of the GE was determined by modifying the procedure outlined by Singleton and Rossi [32]. Briefly, aqueous GE (100 µL) was mixed with 100 µL of freshly prepared 0.2 M FCR and with 100 µL of a 6% *w/v* Na_2_CO_3_ solution, which was added 1 min after the FCR. The absorbance was read at 750 nm (Tecan Infinite 200 Pro M Nano+, Männedorf, Switzerland) after a reaction time of 60 min in the dark at 25 °C. Serial dilutions of GA (20, 40, 60, 80, 100 mg/L) were used for the making of the standard curve in the same way as described above (y = 4.0704x − 0.0338; R^2^ = 0.9999). The results were expressed as milligrams of GAE per milliliter of extract (mg GAE/mL extract). The application of GE for testing antibacterial effects was performed with the fresh extract, following the storage at −20 °C, after which TPC was determined.

In addition to the OMWW samples and GE sample, the potential antibacterial effects of the phenol hydroxytyrosol (HTyr, Sigma-Aldrich, Merck, Darmstadt, Germany) in concentrations of 2.5 and 1.25 mg/mL was assessed against *P. savastanoi* pv. *savastanoi* as a reference and control treatment, alongside the standard antibiotic tetracycline (AB) at a concentration of 0.5 mg/mL and the copper-based preparation Nordox 75 WG (Cu_2_O) at a concentration of 2.0 mg/mL (Table 1). 

### 2.5. Qualitative Determination of Antibacterial Effect on Bacterial Inoculum

To test antimicrobials (Table 1) for their antibacterial efficacy against *P. savastanoi* pv. *savastanoi,* the Kirby–Bauer disc diffusion protocol was followed [33]. Bacterial density was adjusted to approximately 10^8^ CFU/mL using the 0.5 McFarland Turbidity standard (Remel, Lanexa, KS, US). Prior to antimicrobials’ application, suspensions were thoroughly vortexed and pipetted on discs. The antimicrobials’ efficacy was assessed by applying 15 µL of antimicrobial samples on the discs, followed by incubation at +4 °C for two hours for diffusion, and further incubation at 27 °C for 24 h in the dark. The diameter of the inhibition zones was measured in millimeters using a caliper.

### 2.6. Quantitative Determination of Antibacterial Effect 

MIC values of the chosen antimicrobials were determined after 24 h of incubation based on the difference in the OD_600_ reading of bacterial suspensions with and without treatment. The treatments evaluated for their potential antibacterial effect against *P. savastanoi* pv. *savastanoi* consisted of a series of two-fold serially diluted treatments, with concentrations starting from ½ of the total phenolic content as the determining factor for the potential antibacterial effect (Table 1). 

To compare the antibacterial potential of tested antimicrobials versus the non-treated control, the percentage of bacterial growth inhibition at MIC values was calculated using the following formula:

Inhibition (%) = [(A_600_ − B_600_)/A_600_] × 100

where A_600_ represents the absorbance of the control (bacterial culture without antimicrobial treatment) and B_600_ represents the absorbance of the culture with tested antimicrobial at a specific dilution. Absorbance readings were obtained using a TECAN Infinite 200 PRO spectrophotometer (Männedorf, Switzerland).

### 2.7. Disease Evaluation and Data Analysis

To assess the virulence of each tested *P. savastanoi* pv. *savastanoi* strain, the surface area of knots (mm^2^) per olive variety was considered the determining factor. 

The level of susceptibility of the tested olive varieties to olive knot disease was determined based on the measurements of ellipsoid formations (mm), considering both knot width and length. These measurements were conducted using a digital caliper. 

**Figure 4:**
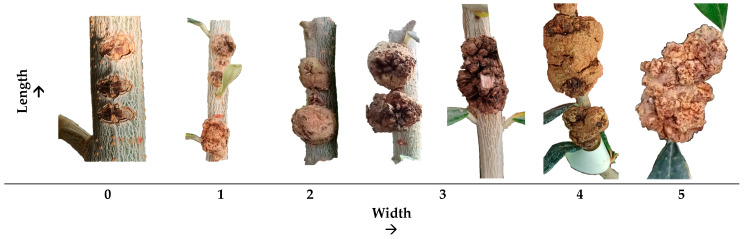
Symptoms caused by experimental inoculation of bacterium *P. savastanoi* pv. *savastanoi* based on the measured parameters, namely the width and length of knots. The numbers below pictures represent the clusters based on both width and length values for the determination of olive cultivar susceptibility to olive knot disease: **0**: no knots; **1**: 1–5.5 mm; **2**: 5.6–10.5 mm; **3**: 10.6–15.5 mm; **4**: 15.6–20.5 mm; and **5**: >20.5 mm.

The measurements were subjected to a slightly adjusted scale derived from Abuamsha et al. [12], utilizing grades ranging from 0 to 5 representing the following clusters: 0—no knots-resistant; 1: 1–5.5 mm—very low susceptibility; 2: 5.6–10.5 mm—low susceptibility; 3: 10.6–15.5 mm—intermediate susceptibility; 4: 15.6–20.5 mm—high susceptibility; and 5: >20.5 mm—very high susceptibility (Figure 4).

For the assessment of effects of treatments and strain-specific responses, the data on the *P. savastanoi* pv. *savastanoi* virulence and olive susceptibility experiment were subjected to two-way ANOVA using the statistical software STATISTICA 12.0 (StatSoft Inc., Tulsa, OK, USA). The differences in susceptibility of five olive cultivars to five *P. savastanoi* pv. *savastanoi* strains were determined for treatments considering the *P. savastanoi* pv. *savastanoi* strain as one factor and the olive cultivar as the second factor. Significant variations in means were determined using Tukey’s post hoc HSD test based on a *p*-value threshold of ≤0.05. Additionally, *t*-tests were used to assess the significance of differences in the percentage of bacterial growth inhibition at MIC values compared to the negative control without treatments.

## 3. Results

### 3.1. Greenhouse Experiment

We performed an evaluation of *P. savastanoi* pv. *savastanoi* strain virulence and the susceptibility of Croatian autochthonous olive cultivars to olive knot disease.

Table 2 presents the results of olive cultivar susceptibility to olive knot disease and the virulence of strains used for the infection of olive trees, based on developed knot length, width, and surface area. 

The two-way ANOVA revealed significant differences in knot width, length, and surface area among five olive cultivars inoculated with five different strains of *P. savastanoi* pv. *savastanoi*. The analysis demonstrated that knot dimensions depend on both the specific strain and the olive cultivar (Table 2). The strain had a significant impact on knot length, while the cultivar influenced knot width more prominently. In general, based on the average width and length of knots, all cultivars exhibited a low level of susceptibility to olive knot disease, except for Rosinjola, which displayed a higher level of susceptibility (cluster 3 and 4, respectively), defined as intermediate susceptibility in Abuamsha et al. [12], compared to other cultivars. 

The most severe olive knot disease, in terms of knot width, was caused by the strain CRO I7L, with an average knot width of 12.07 mm across all cultivars. The strains CFBP5075, CRO B45C-PR, and SLO A1-1 generally produced smaller knots. The cultivars Istarska bjelica and Buža developed the smallest knots across all tested strains. In contrast, cv. Rosinjola was the most susceptible, regardless of the strain. Cv. Frantoio showed high susceptibility to the strain CRO I7L, and cv. Leccino was similarly susceptible to CRO P15N. Leccino exhibited tolerance similar to cv. Istarska bjelica to the strains CFBP5075 and CRO B45C-PR. 

Knot length showed more complex variations among strains and cultivars. All cultivars were least affected by the strain CRO B45C-PR. Cv. Istarska bjelica showed the lowest susceptibility to all tested strains except for CRO I7L, which significantly differed from others in knot length. The cultivars Buža and Leccino also exhibited lower susceptibility to most strains. As with knot width, cv. Rosinjola had the highest knot length values across all strains. The most severe infections were caused by the strains CRO I7L and SLO A1-1, followed by CRO P15N, which did not differ significantly. The highest average knot lengths were observed for the strains CRO I7L and CFBP5075, particularly in cultivars Rosinjola and Leccino. The strain B45C-PR was the least virulent across all cultivars, yet even this strain produced significantly larger knots on cv. Rosinjola compared to other cultivars, similar to the reference strain.

For surface area, the strain CRO I7L was the most virulent, while the strain B45C-PR was the least. All strains severely affected cv. Rosinjola compared to other cultivars. Cv. Istarska bjelica was the least affected variety based on the extent of symptoms, while other cultivars, except for Rosinjola, did not show statistically significant differences when inoculated with B45C-PR. Cv. Leccino was the second most affected, followed by Buža and Frantoio.

**Figure 5:**
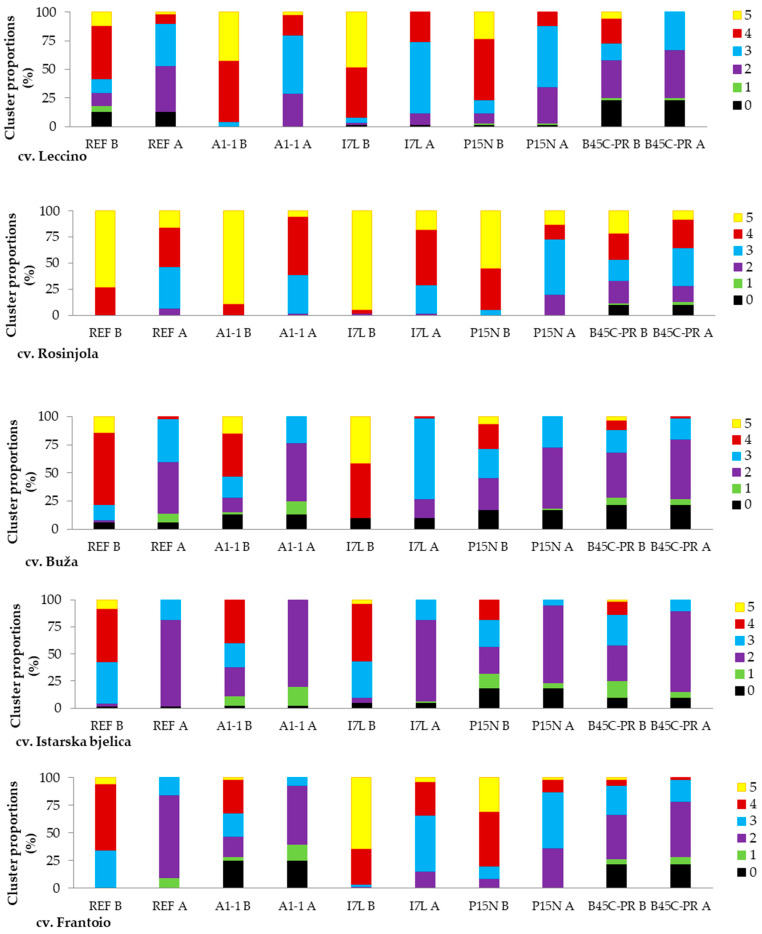
Pathogenicity of five strains of *P. savastanoi* pv. *savastanoi* (reference strain CFBP5075 from Italy, Slovenian strain A1-1, Croatian strains CRO I7L, P15N and B45C-PR) based on the frequency of occurrence and assessment of the magnitude of the symptoms of the disease. Knots’ width, A, and length, B, are indexed in clusters from 0 to 5 (legend) according to a slightly adjusted version of the scale presented by Ambuasha et al. [12]: 0: no knots; 1: 1–5.5 mm; 2: 5.6–10.5 mm; 3: 10.6–15.5 mm; 4: 15.6–20.5 mm; and 5: >20.5 mm. Letter A—knot width; B—knot length.

The analysis of knot characteristics, which involved the distribution of indexed knots based on their size in millimeters and subsequent clustering according to a slightly adjusted version of the scale presented by Ambuasha et al. [12], facilitated the determination of the proportion of each cluster for each parameter (Figure 5). In Figure 5, the proportion of indexed knots was determined as the number of knots representing each cluster out of the total number of knots measured (60 measurements) per strain × cultivar. 

Specifically, for cv. Leccino, over 50% of knots primarily belonged to higher-length clusters (4 or 5) for all *Pss* strains except for the strain B45C-PR. For this strain, the highest proportion of knots in terms of length was indexed in cluster 2 (32.7%), followed by clusters 0, 4, and 3, with few exceeding 20 mm (cluster 5). Most strains elicited a more uniform knot width, predominantly falling into clusters 2 or 3 based on this parameter. Notably, the reference strain REF, the Slovenian strain A1-1, and Croatian strains I7L and P15N showed the highest share of knots ranging from 15.6 to 20.5 mm in width (cluster 4). Additionally, 13% and 24% of inoculation sites did not develop any symptoms (cluster 0) when infected with the reference and B45C-PR strains, respectively.

For cv. Rosinjola, the majority of formed knots (>50%) exhibited greater length (cluster 5) compared to their width (clusters 3 and 4) for all strains except B45C-PR, which showed an almost equal distribution among clusters 2–5. Similar to observations with cv. Leccino, knots did not develop at 10% of inoculation sites when infected with the strain B45C-PR. 

For cv. Buža, the majority of knots were predominantly clustered in cluster 2 based on their width, except when the plants were infected with the Croatian strain I7L, where over 70% of knots were segregated in cluster 3. Similar to the previous olive cultivars, higher clusters were observed for knot length when olive trees were inoculated with the reference, A1-1, and I7L strains. However, for the strains P15N and B45C-PR, the knots exhibited greater variability in length. Specifically, the strain P15N induced the formation of knots that exceeded 20% across clusters 2–4, with 17% of inoculation sites resulting in the absence of knots. The strain B45C-PR had the highest proportion of knots developing in cluster 2 length (40%), with 22% exhibiting no symptoms (cluster 0). 

Knots developing at inoculation sites on cv. Istarska bjelica resulted in over 70% being clustered in cluster 2 for width and over 40% being clustered in cluster 4 for length. Similar to observations with cv. Buža, the strains P15N and B45C-PR displayed deviations from this pattern regarding knot length. Specifically, knots exhibited smaller widths and were equally nested in clusters 2 and 3 at 25%, while the proportion of knots in these clusters for the strain B45C-PR was 33% and 28%, respectively. The highest proportion of asymptomatic sites was observed with P15N inoculation (18%), followed by B45C-PR (10%).

Moreover, knots developed on cv. Frantoio exhibited the highest variability in measured parameters, with the majority segregating into numerous clusters, highlighting the cultivar’s susceptibility to olive knot disease. Generally, the distribution of knots for the reference strain, A1-1, and B45C-PR was comparable, with approximately 75%, 53%, and 50% nested in cluster 2 based on their width, and with proportions of 60% and 30% in cluster 4 based on length. Infection with the Slovenian strain A1-1 and B45C-PR resulted in 25% and 22% of sites being non-symptomatic, respectively. Conversely, for the two Croatian strains (I7L and P15N), knots were predominantly characterized by cluster 3 for width and clusters 4 and 5 for length.

### 3.2. Antibacterial Efficacy of Plant-Based Antimicrobials

#### 3.2.1. Qualitative Characterization

The results presented in Table 3 indicate the variations in the antibacterial effect of tested agents against three strains of *P. savastanoi* pv. *savastanoi*, as determined by the disc diffusion susceptibility test. The effectiveness of the treatments was assessed based on the size of the clearing zone (halo) around the disc. In summary, 3 out of the 13 test treatments demonstrated moderate antibacterial efficiency when compared to the effectiveness of standard antibiotic and copper-based preparations. The largest clearing zones, exceeding 16 mm, were observed for the treatment with aqueous fresh GE, indicating its comparable effectiveness to the copper-based treatment. In other treatments, lower inhibition of bacterium was determined solely for HTyr. The application of OMWW samples showed no inhibition against *P. savastanoi* pv. *savastanoi* strains. 

#### 3.2.2. Evaluation of Antibacterial Effect and MICs of Plant-Based Preparations

**Figure 6:**
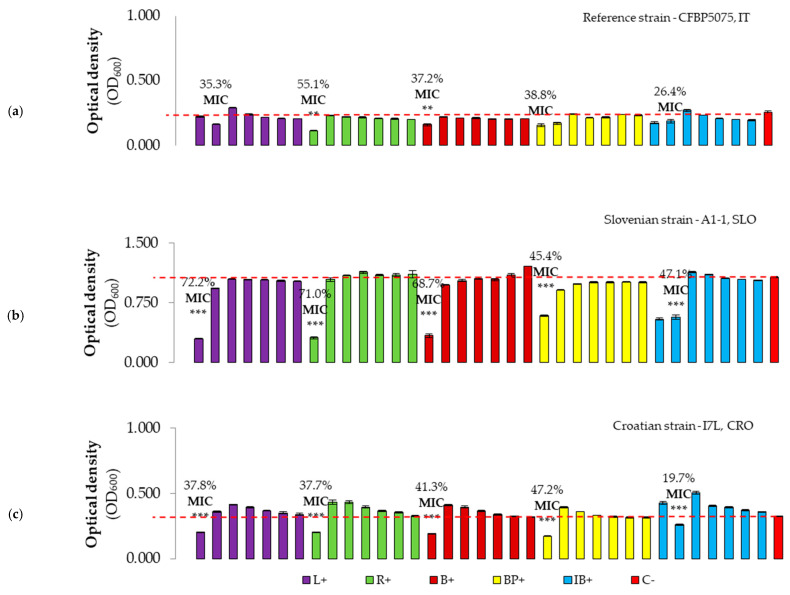
Antibacterial effect and MIC of olive mill wastewater treatments with pH adjusted to 2.0 with HCl against three strains of the bacterium *P. savastanoi* pv. *savastanoi*, namely (**a**) the reference strain CFBP5075, Italy; (**b**) the Slovenian strain A1-1, Slovenia, Istria; and (**c**) the Croatian strain I7L, Croatia, Istria. OD_600_ values of each antimicrobial with the bacterium are presented in different colors, with each color representing one treatment with seven concentrations (C1 to C7, from left to right). The red bars (C-) represent the negative control (bacterium without treatment). The y axis represents the optical density (600 nm) value, while the x axis shows the serially diluted concentrations of the treatments used. The numbers above the bars represent the percentage of inhibition of bacterial growth for the most pronounced treatments. Asterisks indicate the difference in MIC compared to the negative control with a significance level of *p* ≤ 0.01 (**), *p* ≤ 0.001 (***).

**Figure 7:**
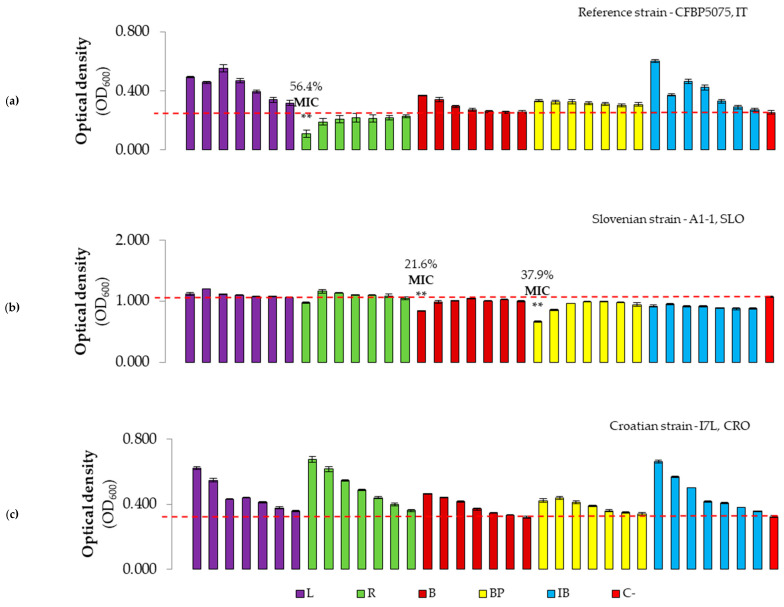
Antibacterial effect and MIC of olive mill wastewater treatments with non-adjusted pH against three strains of the bacterium *P. savastanoi* pv. *savastanoi*, namely (**a**) the reference strain CFBP5075, Italy; (**b**) the Slovenian strain A1-1, Slovenia, Istria; and (**c**) the Croatian strain I7L, Croatia, Istria. OD_600_ values of each antimicrobial with bacterium are presented in different colors, with each color representing one treatment with seven concentrations (C1 to C7, from left to right). The red bars (C-) represent the negative control (bacterium without treatment). The numbers above the bars represent the percentage of inhibition of bacterial growth at MIC values of treatments. The y axis represents the optical density (600 nm) values, while the x axis shows the serially diluted concentrations of treatments used for the experiment. Asterisks indicate the difference in MIC compared to the negative control with a significance level of *p* ≤ 0.01 (**).

**Figure 8:**
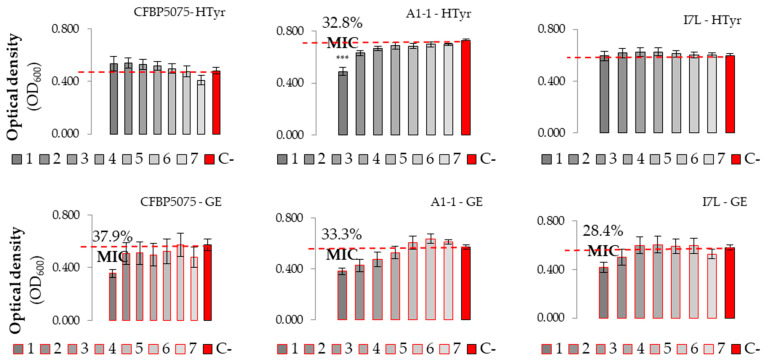
Antibacterial effect of HTyr and GE treatments against three strains of the bacterium *P. savastanoi* pv. *savastanoi*. OD_600_ values of each antimicrobial with bacterium are presented in different shades, each representing one treatment with seven concentrations (C1 to C7). The red bars (C-) represent the negative control (bacterium without treatment). The y axis represents the optical density (600 nm) values, while the x axis shows the serially diluted concentrations of treatments used for the experiment. The numbers above the bars represent the percentage of inhibition of bacterial growth at MIC values of treatments. Asterisks indicate the difference in concentrations compared to the negative control with a significance level of *p* ≤ 0.001 (***).

To compare the antibacterial effect of the tested antimicrobials versus the non-treated control, the percentage of bacterial growth inhibition values were determined (Figure 6, Figure 7 and Figure 8). The highest inhibition of 72.2% was exerted by the first concentration of L+ against the reference strain, whilst the lowest inhibition rate of 19.7% was determined for the second dilution of IB+ against the Croatian *Pss* strain (Figure 6). In general, the MIC was observed for the highest concentration of treatments, except for the second concentration (C2, second column) of L+ against the reference strain. Similar results were obtained for IB+ OMWW, where the MIC was also observed for C2 (0.186 mg GAE/mL) against all strains tested. MIC values were significantly lower compared to the negative control treatment for the Slovenian and Croatian strains, although this trend was not consistently observed for the reference strain, except for the MIC of B+ and R+ OMWW. 

When analyzing OMWWs without hydrochloric acid (HCl) (Figure 7), the MIC was only determined for the R treatment against the reference strain, and for B and BP OMWWs against the Slovenian strain of *Pss*. These MIC values were significantly lower compared to the negative control treatment. Overall, the antibacterial effect of OMWWs was low or absent. 

The antibacterial efficacy of the phenol HTyr exhibited a low impact on all three strains of *Pss* in the range of tested concentrations (Figure 8). Only the Slovenian strain A1-1 showed a statistically significant reduction in absorbance values at the highest tested concentration (2.5 mg/mL), indicating an MIC. Similar outcomes were observed when assessing the antibacterial potential of GE. MIC values were determined for all strains. Furthermore, statistically, the MIC of GE did not differ from the negative control for any *P. savastanoi* pv. *savastanoi* strain.

## 4. Discussion

The control of *P. savastanoi* pv. *savastanoi* remains a challenge as its spread and virulence are strongly influenced by numerous factors including climatic conditions, olive variety, agro-technical measures, and the complex microbial community within the host [13,14]. Current control measures of olive knot disease primarily rely on pruning and copper-based treatments due to the absence of effective bactericidal preparations. However, the limited availability of bactericidal formulations has spurred the exploration of alternatives, including essential oils, plant extracts, and byproducts from plant processing, such as olive oil production wastes. In this study, the virulence of *P. savastanoi* pv. *savastanoi* strains, the susceptibility of five olive cultivars through experimental inoculation directly to wounds, and the antibacterial potential of some plant-based agents were determined.

Analysis showed differences in susceptibility among the included cultivars to olive knot disease, as well as differences in virulence among the *P. savastanoi* pv. *savastanoi* strains used in the greenhouse experiment. Based on the previous field trials [28], it was expected that Croatian cultivars would exhibit low susceptibility to olive knot disease when inoculated with *P. savastanoi* pv. *savastanoi* in the greenhouse experiment. The hypothesis was confirmed with cv. Istarska Bjelica, which was found to be the most tolerant to olive knot disease, but rejected for cv. Rosinjola, which showed higher susceptibility compared to other cultivars. In general, knot formation exhibited a tendency towards length, with most knots aligning with higher-length clusters across various olive cultivars and *P. savastanoi* pv. *savastanoi* strains. Cv. Frantoio exhibited high variability, with the strains A1-1 and B45C-PR being the least virulent, with the highest share of non-symptomatic inoculation sites indexed as cluster 0 among the tested olive cultivars, while cv. Leccino exhibited small knots with specific strains, notably B45C-PR. The highest share of non-symptomatic wounds was detected for cv. Buža. Interestingly, two Croatian strains resulted in contrasting levels of virulence, where the Croatian strain B45C-PR showed the lowest virulence level and the Croatian strain I7L displayed the highest. The observed differences in *P. savastanoi* pv. *savastanoi* strain virulence could be driven by adaptive evolution to host defense mechanisms or genetic variations among strains. Notably, all strains, including the most virulent strain, I7L, induced the smallest knots on cv. Istarska bjelica, confirming its value as a promising variety for wider cultivation in olive-growing regions where olive diseases are compromising olive production. Notably, during the experimental inoculation, variations were noted in olive twig bark firmness and internode length, with higher levels of bark firmness and smaller internode length observed in the least susceptible cv. Istarska bjelica compared to the others. The determined low susceptibility of cv. Istarska bjelica to olive knot disease aligns with previous field observations, as evidenced by its absence or smaller knot sizes compared to other varieties grown in Croatian and Slovenian orchards [28]. 

Based on the proposed classification of susceptibility to olive knot disease by Ambuamsa et al. [12], it was determined that the cultivars Buža and Frantoio do not differ from the tolerant cv. Istarska bjelica; however, those cultivars showed significantly higher dimensions of the length and surface area of the knots. Furthermore, cv. Rosinjola could be clearly differentiated from other cultivars based on the same parameters. Regarding these findings, we propose that the studied cultivars could be segregated into four clusters based on the surface area, as follows: 1—low susceptibility—Istarska bjelica; 2—intermediate susceptibility—Leccino; 3—high susceptibility—Frantoio and Buža; and 4—very high susceptibility—Rosinjola. Alternatively, we could segregate cv. Frantoio, Leccino, and Buža as intermediately susceptible cultivars, based on length of knots. The latter classification is not in accordance with the literature, where cv. Leccino exhibits significantly higher tolerance than the susceptible cv. Frantoio, suggesting that more reliable observations could be drawn from the surface area parameter. Also, the latest research by Xie et al. [22] on the phenolic profile of different plant tissues of the Leccino and Frantoio olive cultivars could provide insights into the observed variation in susceptibility of olive cultivars. Specifically, their study found that the cv. Leccino exhibited higher concentrations of oleuropein in its branches compared to other tissues, while cv. Frantoio displayed a more abundant and varied chemical composition across most tissues analyzed. Similar results were reported for cv. Leccino from Croatia, where the leaves were the most abundant in hydroxytyrosol; however, the content of phenols in the tested varieties was variable throughout the year, whilst the content in cv. Istarska bjelica was uniform [35]. This possibly suggests that the stability of phenolic content in cv. Istarska bjelica is partially responsible for its highest tolerance in this study. Moreover, cv. Leccino exhibits lower susceptibility to olive knot disease [12,15], as confirmed in a previous field experiment in the Istrian region, where the incidence of olive knot disease on the Leccino variety was determined to be 12% and 28%, depending on the year and copper-based treatment [36]. Also, this is in accordance with the recent study conducted in Dalmatia region in Croatia, where Leccino was classified as the least susceptible olive cultivar compared to others, including four Croatian cultivars [15]. The diversity of susceptibility of Croatian olive genotypes highlights the importance of determining the chemical profile of different plant tissues, given that *Pss* primarily thrives in infections on branches and twigs. Nevertheless, the presence of certain fungal and bacterial species, pathogenic or non-pathogenic, has emerged as a crucial factor influencing susceptibility to olive knot disease in diverse olive cultivars worldwide [14], a phenomenon yet to be extensively studied in Croatian varieties, but could be a crucial factor for the determined low susceptibility of cv. Istarska bjelica used in our study. Notably, it is determined that the susceptible olive cultivars to olive knot disease and olive quick decline syndrome caused by the bacterium *P*. *savastanoi* pv. *savastanoi* and *Xylella fastidiosa* are more abundant in fungal communities such as *Alternaria*, *Neofusicoccum*, *Epicoccum*, *Phaeoacremonium*, etc. [8,14,37], which were recently reported as emerging pathogens in Istrian olive orchards in Croatia [38,39]. 

The process of characterization of the OMWWs encompassed the determination of the pH value and TPC, with the purpose of obtaining the simultaneous coverage of a larger range of phenol concentrations and possible differences in the phenol profile, which may be different in different varieties. A higher pH value is required for the reaction of phenol with FCR, so sodium carbonate is added to the analysis to obtain pH = 10 [40]. Although it was assumed that the addition of 0.10–0.25 µL of the acidified OMWW sample would not compromise the reaction, it is possible that this is the reason for the slightly lower phenolic concentrations found in the OMWW samples acidified to pH 2. When the antibacterial properties of the tested plant-based agents are considered, the results of this study revealed low to no efficacy of OMWWs against *P. savastanoi* pv. *savastanoi* strains, contrary to some previous studies [25,30,41]. This lack of effectiveness could be attributed to factors such as the bacterial degradation of phenolic compounds [7,42] or the limited diffusion of treatments in the growth medium during in vitro testing [43,44]. Also, considering that in several treatments, overgrowth occurred, it is possible that due to the complexity in composition of OMWW, the microorganism cells found sugars and other organic compounds as well as microelements from OMWW as the substrate for growth, surpassing the inhibitory effect of phenols. Another reason could be that the phenols degraded during several months of storage, with a consequent reduction in antibacterial potential. 

The growth inhibition of *P. savastanoi* pv. *savastanoi* strains exerted by the antimicrobials tested was generally below 50% at the highest concentrations applied. However, despite the lower TPC in OMWWs B+, BP+, and R+ compared to L+ and IB+, the observed growth inhibition rate of all treatments remains nearly negligible, except against the Slovenian strain A1-1 treated with L+, R+, and B+ reaching 72.2, 71.0, and 68.7% of inhibition of growth, respectively, and the treatments R+ and R against the reference strain, with inhibition of 55% and 56%, respectively. The observed variation in the inhibition of *Pss* growth could be a consequence of differences in virulence and pH susceptibility among tested strains, as the Croatian strain I7L showed lower susceptibility to OMWWs in vitro and the highest virulence in planta. While the antibacterial effect was determined for a concentration of 10% OMW using the agar diffusion test in another study [41], our results showed that pure OMWWs did not exhibit any growth inhibition against *P. savastanoi* pv. *savastanoi* through the disc diffusion method. This discrepancy between studies potentially confirms that OMWWs can exhibit antibacterial properties if their phenolic compounds are highly concentrated in their chemical profile. Moreover, in a recent study [30], the authors investigated the effect of various phases of filtration and distillation treatments as a factor influencing the antibacterial effect of OMW from the olive cv. Taggiasca. In the same study, it was observed that the MICs of antibacterial treatments depended on the TPC, with lower MICs determined against *P*. *syringae* for treatments with higher concentrations, and vice versa, possibly suggesting that the TPCs of OMWWs tested in our study are low in the total phenols that could act as antibacterials. Considering the trend of increasing absorbance, i.e., the number of bacteria, with increasing dilution of OMWW samples, it was assumed that overgrowth occurred when the concentration of phenols was not sufficient to stop the growth of bacteria, while serving as a source of soluble sugars and other nutrients for bacterial growth and development. When polyphenols from OMWW were tested against bacterial species *Xanthomonas campestris* spp., the MIC occurred at a concentration of 2.5 mg/mL of TPC [25], further confirming the low effect of OMWWs investigated in this study. Aligning with the study by Russo et al. [30], the concentrations of total polyphenols of our OMWWs were similar to those obtained by microfiltration (MF2), where the antibacterial effect was not observed. Still, their antibacterial potential needs to be tested in planta, as they significantly inhibited the knot formation in study by Krid et al. [41].

Some studies have suggested that the antibacterial effect of OMWWs is attributed to the presence of specific phenolic compounds, particularly HTyr [27]. Indeed, our results support this with determined antibacterial effect of HTyr with the disc diffusion method, where the higher effect was observed with higher concentrations applied. However, when compared to the copper-based treatment, HTyr exhibited only a moderate effect. Similarly, the application of GE exhibited a nearly equivalent effect to copper-based treatments in a disc diffusion study but low efficiency in microdilution testing, potentially due to the volatile nature and consequent evaporation of plant extracts, specifically allicin as the major sulfur-containing volatile compound, responsible for the antibacterial effect of garlic [45,46]. Another study conducted to determine the antibacterial potential of HTyr and HTyr-enriched OMWW extract showed inhibitory effects against *P. savastanoi* pv. *savastanoi* at concentrations of 1 mg/mL of HTyr alone, resulting in approximately a 2-log reduction in CFU/mL [47]. However, significantly stronger activity was observed for the enriched OMWW extract, where a 100% reduction in *P. savastanoi* pv. *savastanoi* growth was determined, confirming the importance of the synergy of minor phenolic compounds and a higher initial concentration of phenols in the chemical profile for the effectiveness of potential plant-based agents. Consistent with this research, the antibacterial effect of phenols in olive wastewater was observed against *P. savastanoi* pv. *savastanoi* and *Clavibacter michiganensis*, with HTyr showing greater efficacy against the Gram-negative bacterium compared to the Gram-positive bacterium [48]. Despite the observed variations in the antibacterial effect, it can be assumed that OMWWs and their chemical components such as HTyr possess antibacterial properties which highly depend on their concentration in OMWWs or as individual agents. This highlights the further evaluation of combinations of active ingredients in formulations to broaden the spectrum and achieve sufficient control effectiveness in disease management strategies against plant pathogens.

The variations in antibacterial activity of plant-based compounds against different strains of a single bacterial species have been documented in the literature previously. It is hypothesized that these differences among strains may be attributed to the presence of genes conferring resistance to antimicrobials, influenced by the gene plasticity of bacteria [6,49]. This phenomenon might be a consequence of the virulence of specific strains, as supported by Moretti et al. [9], who noted strains of *P. savastanoi* pv. *savastanoi* originating from the east coast of the South Adriatic as less virulent compared to Italian and Portuguese strains. However, our study identified the Croatian strain I7L (north Adriatic), isolated from cv. Leccino, as the most virulent among the tested strains, surpassing the Italian strain CFBP5075, the Slovenian strain A1-1 from Arbequina, and other Croatian strains, namely P15N isolated from Nocciara and B45C-PR from Porečka rosulja. Despite this, identifying low-virulence strains could offer insights into potential microbial-based control strategies for olive knot disease in severely affected orchards. Therefore, these observations underscore the importance of further genetic and phylogenetic research to delineate possible differences in Croatian strains. Moreover, these findings may be linked to the bacterial ability to degrade phenolic compounds [42], which is common among bacterial species within the *P*. *savastanoi* complex, known for expressing virulence through enzymatic interference with host defense mechanisms. This highlights the need for future studies to validate the hypothesis of interaction between bacterial strain virulence, host chemical composition, and geographical origin, thereby elucidating potential genetic variations underlying strain-specific virulence. Notably, the low susceptibility of cv. Istarska bjelica encourages the further investigation of Croatian autochthonous germplasms as valuable tools for the determination of the presence of resistant genes that could be used in future breeding programs.

## 5. Conclusions 

In conclusion, this study provides novel insights into the virulence of *P. savastanoi* pv. *savastanoi* strains, the susceptibility of Croatian autochthonous olive cultivars to olive knot disease, and the in vitro potential of plant-based agents against *P. savastanoi* pv. *savastanoi*. The Croatian strain I7L was identified as the most virulent *Pss* strain, surpassing the virulence of other tested *P. savastanoi* pv. *savastanoi* strains. The diversity in the virulence of strains underscores the complexity of strain-specific interactions with olive cultivars. Indeed, the relationship of virulence diversity of *Pss* strains with geographical origin was determined in previous studies and is confirmed at an even more narrow level in this study. The olive cultivar from a Croatian autochthonous germplasm that showed the highest tolerance to the olive knot disease was Istarska bjelica, surpassing the other tested cultivars. 

Regarding OMWW’s antibacterial effect against *P. savastanoi* pv. *savastanoi* strains suggest the need for further testing with undiluted or even concentrated OMWW samples and exploration of the potential antibacterial effect of concentrates or extracts of phenolic compounds as alternative natural control measures. However, the GE and HTyr showed some antibacterial potential with disc diffusion, and thus should be considered for further evaluation against *P. savastanoi* pv. *savastanoi*. Further studies building upon these findings are essential to develop effective and sustainable preventive strategies against the most persistent bacterial disease of olives, ensuring the constant productivity and greater resilience of olive orchards worldwide.

## Figures and Tables

**Table 1:** The list of antimicrobials used for antibacterial testing against the bacterium *P. savastanoi* pv. *savastanoi.*

Type of Antimicrobial	Antimicrobial	Code	pH	TPC, mg GAE/mL							
				Initial Concentration *	Dilutions **						
					1:2 (C1)	1:4 (C2)	1:8 (C3)	1:16 (C4)	1:32 (C5)	1:64 (C6)	1:128 (C7)
OMWW	Istarska bjelica	IB	5.26	0.757 ± 0.013	0.379	0.189	0.095	0.047	0.024	0.012	0.006
	Leccino	L	6.06	0.327 ± 0.000	0.164	0.082	0.041	0.020	0.010	0.005	0.003
	Rosinjola	R	6.66	0.220 ± 0.014	0.110	0.055	0.028	0.014	0.007	0.003	0.002
	Buža	C	6.00	0.140 ± 0.007	0.070	0.035	0.018	0.009	0.004	0.002	0.001
	Buža puntoža	BP	7.17	0.046 ± 0.002	0.023	0.012	0.006	0.003	0.001	0.001	0.000
OMWW acidified to pH = 2.0	Istarska bjelica	IB+	2.00	0.744 ± 0.017	0.372	0.186	0.093	0.047	0.023	0.012	0.006
	Leccino	L+	2.00	0.252 ± 0.003	0.126	0.063	0.032	0.016	0.008	0.004	0.002
	Rosinjola	R+	2.00	0.156 ± 0.001	0.078	0.039	0.020	0.010	0.005	0.002	0.001
	Buža	B+	2.00	0.101 ± 0.005	0.051	0.025	0.013	0.006	0.003	0.002	0.001
	Buža puntoža	BP+	2.00	0.035 ± 0.000	0.018	0.009	0.004	0.002	0.001	0.001	0.000
Garlic extract	Aqueous extract of Garlic	GE	n.d.	0.064 ± 0.001	0.032	0.016	0.008	0.004	0.002	0.001	0.001
Pure phenol	Solution of Hydroxytyrosol in water	HTyr	n.d.	2.50	2.500	1.250	0.625	0.313	0.156	0.078	0.039
				1.25							
Standard antibiotic	Tetracycline	AB	n.d.	0.50							
Cu-based preparation	Copper (I) oxide (Nordox 75 WG)	Cu_2_O	n.d.	2.00							

n.d.—not determined; initial concentration of OMWW and GE samples represent mean values of two and three analytical determinations, respectively; * used for qualitative testing of antimicrobials efficiency; ** used for quantitative determination of MIC.

**Table 2:** The knot width, length, and surface area following inoculation of five olive cultivars with five different strains of *P. savastanoi* pv. *savastanoi* in a greenhouse experiment. The values presented in the table are expressed as the mean of 60 measurements ± standard error.

		*P. savastanoi* pv. *savastanoi* (S)							
	Olive Cultivar (C)	REF CFBP5075	SLO A1-1	CRO I7L	CRO P15N	CRO B45C-PR	Average per Olive Cultivar	Cluster *	C × S
**Width ** **(mm)**	Frantoio	7.14 ± 0.44 h–j	5.53 ± 0.50 ij	13.85 ± 0.54 a–c	10.68 ± 0.54 d–g	6.98 ± 0.57 h–j	8.84 ± 0.29	2	***
	Leccino	9.50 ± 0.64 f–h	9.58 ± 0.74 e–h	11.47 ± 0.72 c–f	11.53 ± 0.44 c–f	7.02 ± 0.65 h–j	9.82 ± 0.30	2	
	Rosinjola	12.41 ± 0.95 b–f	14.73 ± 0.71 ab	16.11 ± 0.75 a	13.05 ± 0.74 a–d	12.65 ± 0.83 b–e	13.79 ± 0.36	3	
	Buža	7.78 ± 0.57 g–j	7.30 ± 0.49 h–j	10.59 ± 0.52 d–g	7.47 ± 0.53 h–j	7.04 ± 0.55 h–j	8.04 ± 0.25	2	
	Istarska bjelica	7.21 ± 0.46 h–j	5.13 ± 0.36 j	8.34 ± 0.36 g–i	6.13 ± 0.44 ij	6.91 ± 0.38 h–j	6.74 ± 0.19	2	
	Average per *Pss* strain	8.81 ± 0.31	8.45 ± 0.33	12.07 ± 0.31	9.77 ± 0.29	8.12 ± 0.30			
**Length** **(mm)**	Frantoio	14.73 ± 0.85 c–f	10.22 ± 0.94 f–j	21.44 ± 0.59 ab	16.75 ± 0.85 c–e	8.21 ± 0.74 j	14.27 ± 0.45	3	***
	Leccino	14.11 ± 0.95 d–g	15.71 ± 1.17 c–e	16.60 ± 1.06 c–e	17.17 ± 0.65 b–e	8.94 ± 0.93 ij	14.51 ± 0.46	3	
	Rosinjola	17.49 ± 1.30 b–e	22.87 ± 1.05 a	22.57 ± 0.97 a	19.39 ± 0.88 a–c	14.43 ± 1.08 d–f	19.35 ± 0.51	4	
	Buža	13.80 ± 0.94 d–h	14.06 ± 0.92 d–h	17.90 ± 0.81 b–d	10.88 ± 0.92 f–j	8.18 ± 0.75 j	12.96 ± 0.43	3	
	Istarska bjelica	13.03 ± 0.85 e–i	9.62 ± 0.87 g–j	14.89 ± 0.64 c–f	8.65 ± 0.77 ij	9.43 ± 0.68 h–j	11.12 ± 0.37	3	
	Average per *Pss* strain	14.63 ± 0.45	14.50 ± 0.52	18.68 ± 0.41	14.57 ± 0.43	9.84 ± 0.40			
**Surface area** **(mm^2^)**	Frantoio	124.49 ± 9.59 e–g	82.04 ± 10.19 g–i	309.99 ± 17.53 b	203.26 ± 15.65 cd	79.37 ± 9.14 hi	159.83 ± 7.64		***
	Leccino	166.34 ± 15.43 de	197.96 ± 19.13 cd	232.56 ± 17.62 c	212.39 ± 12.63 c	95.21 ± 12.16 f–i	180.89 ± 7.48		
	Rosinjola	285.65 ± 25.65 b	377.97 ± 23.07 a	401.56 ± 23.38 a	287.29 ± 22.48 b	230.67 ± 25.94 c	316.63 ± 11.34		
	Buža	134.54 ± 11.42 ef	126.45 ± 11.43 ef	211.68 ± 11.12 c	105.89 ± 11.25 f–i	78.79 ± 9.26 hi	131.47 ± 5.49		
	Istarska bjelica	115.02 ± 9.16 f–h	65.42 ± 6.98 i	136.15 ± 8.35 ef	70.65 ± 7.33 i	77.91 ± 7.49 hi	93.03 ± 3.86		
	Average per *Pss* strain	165.21 ± 7.78	169.97 ± 9.48	258.39 ± 9.00	175.90 ± 7.96	112.39 ± 7.29			

Different lowercase letters indicate significant differences of knot width, length, and surface area among five olive cultivars inoculated with five different strains of the bacterium *P. savastanoi* pv. *savastanoi* based on the *p* value ≤ 0.05; * classification of olive cultivars’ susceptibility to olive knot disease based on a slightly adjusted version of the scale presented by Abuamsha et al. [12]. Asterisks indicate the significance of the interaction (two-way ANOVA) of olive cultivar × *P. savastanoi* pv. *savastanoi* strain at the level of *p* ≤ 0.001 (***).

**Table 3:** Antibacterial efficacy of tested plant-based and reference antimicrobial preparations based on growth inhibition area (in mm) against three different strains of the bacterium *Pseudomonas savastanoi* pv. *savastanoi* using the disc diffusion method (results are represented by the mean value of nine disc measurements ± standard deviation).

Group of Treatments	Treatment	Growth Inhibition of *P. savastanoi* pv. *savastanoi* Strains (Clearing Zone, mm)		
		REF CFBP5075	SLO A1-1	CRO I7L
OMWWs	non-acidified and acidified	0.00 ± 0.00 ^x^	0.00 ± 0.00 ^x^	0.00 ± 0.00 ^x^
Phenol	*Hydroxytyrosol* (HTyr): 2.5 mg/mL	11.64 ± 3.15 ^+^	10.13 ± 1.12 ^+^	11.26 ± 1.60 ^+^
	1.25 mg/mL	7.03 ± 2.32 ^+^	9.19 ± 0.92 ^+^	9.57 ± 1.14 ^+^
Aqueous extract	Garlic (GE)	16.17 ± 2.36 ^+^	16.30 ± 1.85 ^+^	17.80 ± 1.71 ^+^
Copper-based Treatment	Copper (I) oxide	19.00 ± 0.85	18.81 ± 1.03	17.84 ± 2.93
Antibiotic	Tetracycline	25.49 ± 0.84	25.45 ± 1.17	25.57 ± 0.66

Efficiency evaluated using an adjusted scale of Aires et al. [34]. ^x^—non-effective; ^+^—moderate efficacy (clearing zone less than antibiotic and copper).

## Data Availability

Data are contained within this article.
